# Comprehensive proteomic analysis of white blood cells from chikungunya fever patients of different severities

**DOI:** 10.1186/1479-5876-12-96

**Published:** 2014-04-11

**Authors:** Nitwara Wikan, Sarawut Khongwichit, Weerawat Phuklia, Sukathida Ubol, Tipparat Thonsakulprasert, Montri Thannagith, Duangrudee Tanramluk, Atchara Paemanee, Suthathip Kittisenachai, Sittiruk Roytrakul, Duncan R Smith

**Affiliations:** 1 Institute of Molecular Biosciences, Mahidol University, Salaya Campus 25/25 Phuttamonthol Sai 4, Nakorn Pathom 73170, Thailand; 2 Department of Microbiology, Faculty of Science, Mahidol University, Bangkok, Thailand; 3 Center for Emerging and Neglected Infectious Diseases, Mahidol University, Bangkok, Thailand; 4 Community Medical Unit, Pang Nga Hospital, Pang Nga, Thailand; 5 National Center for Genetic Engineering and Biotechnology (BIOTEC), National Science and Technology Development Agency, Pathum Thani, Thailand

**Keywords:** Chikungunya, Proteome, Inflammasome, Caspase 1

## Abstract

**Background:**

Chikungunya fever (CHIKF) is a recently re-emerged mosquito transmitted viral disease caused by the chikungunya virus (CHIKV), an *Alphavirus* belonging to the family *Togaviridae*. Infection of humans with CHIKV can result in CHIKF of variable severity, although the factors mediating disease severity remain poorly defined.

**Methods:**

White blood cells were isolated from blood samples collected during the 2009-2010 CHIKF outbreak in Thailand. Clinical presentation and viral load data were used to classify samples into three groups, namely non chikungunya fever (non-CHIKF), mild CHIKF, and severe CHIKF. Five samples from each group were analyzed for protein expression by GeLC-MS/MS.

**Results:**

CHIKV proteins (structural and non-structural) were found only in CHIKF samples. A total of 3505 human proteins were identified, with 68 proteins only present in non-CHIKF samples. A total of 240 proteins were found only in CHIKF samples, of which 65 and 46 were found only in mild and severe CHIKF samples respectively. Proteins with altered expression mapped predominantly to cellular signaling pathways (including toll-like receptor and PI3K-Akt signaling) although many other processes showed altered expression as a result of CHIKV infection. Expression of proteins consistent with the activation of the inflammasome was detected, and quantitation of (pro)-caspase 1 at the protein and RNA levels showed an association with disease severity.

**Conclusions:**

This study confirms the infection of at least a component of white blood cells by CHIKV, and shows that CHIKV infection results in activation of the inflammasome in a manner that is associated with disease severity.

## Background

Chikungunya fever (CHIKF) is a mosquito transmitted viral disease caused by the chikungunya virus (CHIKV), an *Alphavirus* of the family *Togaviridae* that is transmitted by mosquitoes of the *Aedes* genus, principally *A. aegypti* and *A. albopictus*[1]. The genome is a positive sense single stranded RNA of approximately 11.8 kb that encodes two open reading frames. The first open reading frame encodes the non-structural proteins (nsP1 to nsP4) required for replication of the virus, while the second open reading frame encodes the structural proteins (C, E1 and E2) and two small peptides (E3 and 6 k) of uncertain function [2].

Although CHIKV has long been circulating at low levels in many African and Asian countries [3,4], CHIKV recently explosively re-emerged in many countries around the Indian Ocean, notably in India, leading to millions of cases of infection [2,5], and autochthonous transmission was reported in Italy [6] and France [7] and more recently in the Caribbean [8]. CHIKF is normally characterized by fever, headache, rash, myalgia and arthralgia which resolves in a few days or weeks, but occasionally the disease is associated with prolonged (months or years) arthralgia and neurological complications [2,5].

A number of cellular targets of CHIKV have been implicated in the disease process including epithelial, endothelial and fibroblast cells [9], muscle satellite cells [10], cells of the joint synovium [11] as well as immune cells such as monocytes [12] and macrophages [13], and the virus is believed to enter cells through a process of receptor mediated [14] clathrin-independent but Eps-15 dependent endocytosis [15].

CHIKV infection of mammalian cells results in the induction of apoptosis [16-18], a process that may facilitate the spread of the virus to other tissues or organs while evading immune clearance through the presence of the virus in apoptotic blebs [16], although the onset of apoptosis is delayed by the induction of autophagy in the host cell [19]. The induction of autophagy is believed to facilitate virus replication [20,21], as has been proposed with a number of other viruses including dengue virus [22,23]. The induction of these and other pathways is associated with the altered expression of a large number of proteins, and several studies have investigated alteration in the cellular proteome in either cell culture or in an animal model system [24-26]. In the earliest study, Dhanwani and colleagues [25] used a mouse model system and 2D-gel electrophoresis and identified 35 differentially expressed proteins in liver and 15 in brain, with proteins predominantly belonging to stress, inflammation, apoptosis and energy metabolism. Using a similar methodology Thio and colleagues [26] identified 50 differentially regulated proteins predominantly associated with mRNA processing, translation and energy production and cellular metabolism in CHIKV infected WRL-68 (human hepatic Hela derived) cells. In a study using the more sensitive technique of GeLC-MS/MS, Abere and colleagues [24] identified some 90 differentially regulated proteins of diverse cellular pathways. However, none of these studies have investigated the changes occurring in clinical materials. In this study we determined alterations in the proteome of white blood cells isolated from acute phase chikungunya patients suffering from different disease severities.

## Methods

### Ethics statement

This study was approved by the Mahidol University Institutional Review Board (COA.NO.MU-IRB 2010/251.3018) and by the Ethics Review Board of Pang Nga Hospital and written informed consent was obtained from all participants.

### Sample collection and preparation

Patients were classified into 3 groups which were non-chikungunya fever (non CHIKF), mild CHIKF and severe CHIKF as previously described and includes samples from patients described previously [27]. After centrifugation to obtain plasma, red blood cells were eliminated by using red cell lysis buffer. White blood cell (WBCs) pellets were collected after centrifugation and the supernatant removed before storage at -80°C. For protein isolation cell pellets were resuspended in sterile distilled water and sonicated six times for five minutes and proteins precipitated with acetone before centrifugation at 9,200 × g for 30 minutes. The pellets were resuspended in 0.5% SDS and the protein concentration was determined using Bradford reagent (Bio-Rad, Hercules, CA). A total of 20 μg of protein from 5 samples per group (non CHIKF), mild CHIKF and severe CHIKF) were separated by 12.5% SDS-PAGE. Gels were stained with colloidal coomassie blue and each lane was cut into 13 slices according to the size of the separated proteins. Each gel slice was cut into 1 mm^3^ and the proteins inside the gel plugs were subjected to tryptic digestion as previously described [24].

### LC MS/MS

After tryptic digestion, the dry samples were dissolved in 12 μl/well of 0.1% formic acid in LC-MS grade water and analysis of tryptic peptides was performed using a SYNAPT^TM^ HDMS mass spectrometer (Waters Corp., Manchester, UK). For all measurements, the mass spectrometer was operated in the V-mode of analysis with a resolution of at least 10,000 full-width half-maximum. All analyses were performed using the positive nanoelectrospray ion mode. The time-of-flight analyzer of the mass spectrometer was externally calibrated with [Glu^1^]fibrinopeptide B from m/z 50 to 1600 with acquisition lock mass corrected using the monoisotopic mass of the doubly charged precursor of [Glu^1^]fibrinopeptide B. The reference sprayer was sampled with a frequency of 20 sec. Accurate mass LC-MS data were acquired with data direct acquisition mode. The energy of trap was set at a collision energy of 6 V. In transfer collision energy control, low energy was set at 4 V. The quadrupole mass analyzer was adjusted such that ions from m/z 300 to 1800 were efficiently transmitted. The MS\MS survey is over range 50 to 1990 Da and scan time was 0.5 sec.

### Protein quantitation and identification

For proteins quantitation, DeCyder MS Differential Analysis software (DeCyderMS, GE Healthcare) was used and data from DeCyderMS were submitted to database search using the Mascot software (Matrix Science, London, UK) as described previously [24]. The data was searched against the NCBI database for protein identification. Database interrogation was; taxonomy (*Homo sapiens* or *Alphavirus*); enzyme (trypsin); variable modifications (carbamidomethyl, oxidation of methionine residues); mass values (monoisotopic); protein mass (unrestricted); peptide mass tolerance (1.2 Da); fragment mass tolerance (±0.6 Da), peptide charge state (1+, 2+ and 3+) and max missed cleavages (3). The maximum value of each group was used to determine the presence or absence of each identified protein.

### Quantitative real time PCR

Total RNA was extracted from white blood cells obtained from non-CHIKF febrile disease (n = 2), mild CHIKF (n = 3) and severe CHIKF (n = 2) patients using TRI Reagent® (Molecular Research Center, Inc., Cincinnati, OH). Samples were treated with Dnase I (Promaga, Madison, WI) to remove genomic DNA before cDNA generation using oligo-dT (Bio Basic, Inc.) and Improm-II™ reverse transcriptase enzyme (Promega). The generated cDNA was use as template for quantitative real time PCR performed based on the SYBR system using thekAPA SYBR® FAST qPCRkit 2× Master Mix (Kapa Biosystems Inc.*,* Woburn*,* MA) in a Mastercycler® ep realplex real-time PCR system (Eppendorf AG, Hamburg, Germany). Synthesis was carried out at an initial 95°C for 3 min followed by denaturation at 95°C for 10 seconds, annealing at 60°c for 30 seconds and extention at 72°C for 20 secs for 40 cycle using primers for caspase 1 (caspase-1fw: 5′-ACCAGGAAACGGAAACAGAGTGGT-3′ and (caspase-1rv: 5′-CTGCCCACAGACATTCATACA-3′) and actin (Actinfw :5′-ACCAACTGGGACGACATGGAGAAA-3′) and (Actinrv: 5′-TAGCACAGCCTGGATAGCAACGTA-3′). The relative expression levels of caspase 1 mRNA was normalized to actin using the comparative C_T_ method (2^-∆CT^ method). The fold change in expression between the non-CHIKF patients and CHIKF patients was calculated as 2^-∆CT^ (CHIKF patients)/ 2^-∆CT^ (non-CHIKF patients).

## Results and discussion

Whole white blood cells were collected from 5 febrile non-CHIKF patients, 5 mild and 5 severe CHIKF patients. Samples were collected from a subset of patients examined for viral load by qRT-PCR as previously reported [27]. The samples used in this study came from patients with viral loads of log_10_ 8.6 +/- 1.17 and log_10_ 8.9 +/- 1.17 for mild and severe respectively, which was in close agreement with the values of log_10_ 8.3 +/- 1.1 and log_10_ 8.53 +/- 0.9 for mild and severe CHIKV patients respectively as previously reported [27].

Proteins were isolated and separated by one-dimension 12.5% SDS-PAGE and each lane was cut into 13 slices (See Additional file 1: Figure S1). The individual slices were diced into 1 mm^3^ portions and proteins subjected to in gel tryptic digestion. The resulting peptides were analyzed by MS/MS and the resulting data was analyzed with DeCyder MS Differential Analysis software and submitted to database search using the Mascot program.

**Figure 1:**
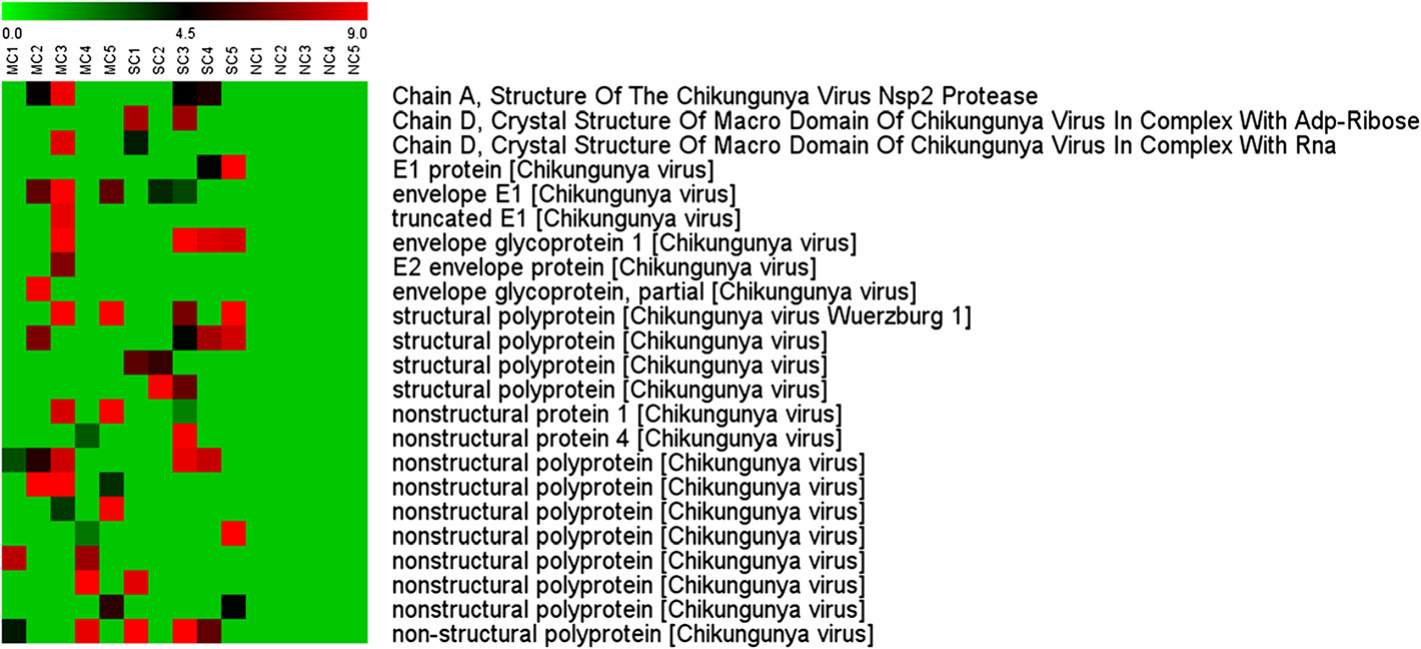
**Hierarchical clustering analysis of differentially expressed Alphavirus proteins.** Peptides identified after GeLC-MS/MS were searched against the Mascot *Alphavirus* database. CHIKV proteins were identified only either in mild CHIKF (MC) or severe CHIKF (SC) samples but not in detected in non CHIKF (NC). Each lane represents one sample from one patient. The color scale is shown by the bar at the top.

The data was initially searched against the Mascot *Alphavirus* database and multiple matches to *Alphavirus* polyprotein and individual structural and non-structural proteins were detected. The data was further sub-selected to highlight matches to CHIKV proteins as shown in Figure 1, and, importantly, matches were limited to cases of mild and severe CHIKF. As shown in Additional file 2 peptide mass matches were found to peptides for all four non-structural proteins. The matches with non-structural proteins supports that at least a subset of the cells are permissive for viral replication as non-structural proteins do not comprise any part of the CHIKV virion, and additionally supports studies that have shown monocytes to be a target cell for CHIKV [12].

A Mascot search of the *Homo sapiens* database resulted in a total of 12,467 peptides which mapped to 3505 unique proteins. Of these 3505 proteins, 514 were present in all samples analyzed, and no protein was found to be differentially regulated with 100% concordance (i.e. down regulated in all non-CHIKF samples and up regulated in all CHIKF samples or vice versa). In total 2886 proteins were present in at least one sample in each of the three sample groups.

**Figure 2:**
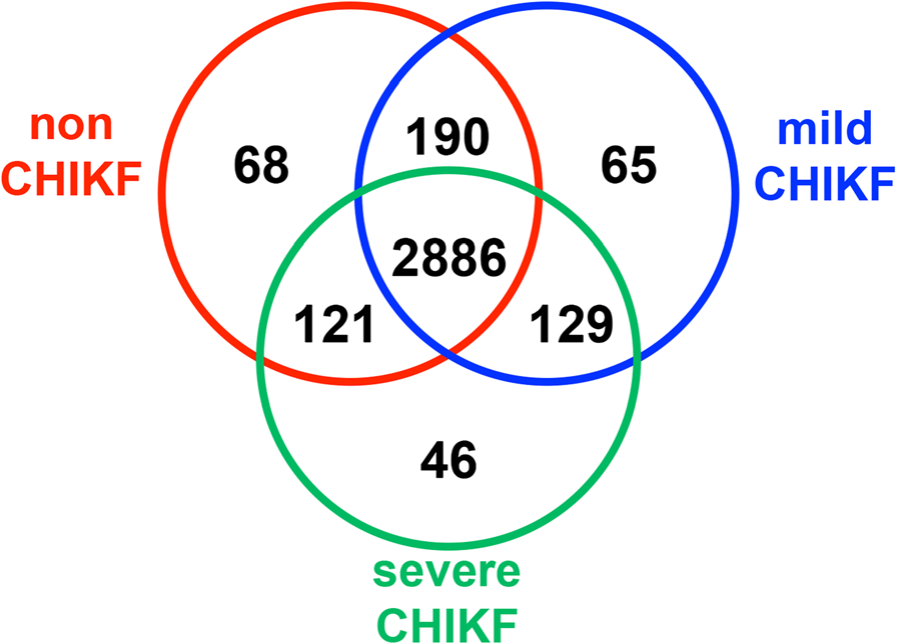
**Summary of significant proteins of WBC samples.** Peptides identified after GeLC-MS/MS were searched against the Mascot *Homo sapiens* database. A total of 3505 proteins were indentified, with 68, 65 and 46 proteins detected only in non CHIKF, mild CHIKF and severe CHIKF, respectively. A total of 240 proteins were identified in CHIKF samples.

Using the criteria of not present in all non-CHIKF samples and present in at least 1 CHIKF sample, at total of 240 proteins were identified, with 65 being detected only in mild CHIKF and 46 only in severe CHIKF samples (Figure 2). Using the criteria of absent in all CHIKF samples and present in at least 1 non-CHIKF sample, 68 proteins were identified (Figure 2).

The 68 proteins found only in non-CHIKF cases are proteins that are down-regulated in response to CHIKF (Additional file 3) as compared to non-CHIKF fever patients. These include extracellular matrix and cytoskeleton associated proteins (collagen alpha-1, laminin subunit alpha-3, protocadherin, WAS protein) as well as those involved in signal transduction (mitogen-activated proteinkinase 8, interacting protein 1, serine/threonine-proteinkinase Sgk1 isoform 4, Voltage dependent calcium channel gamma 3 subunit) and mRNA processing and regulation (60S ribosomal protein L36, DEAD box polypeptide 17, kIAA0020). Down regulation of mRNA processing and regulation proteins in infected cells would be consistent with studies that have shown that CHIKV infection results in both transcriptional and translational shut off through the action of either nsP2 or CHIKV capsid protein [28,29] and down regulation of extracellular matrix proteins could occur as a result of either cell remodeling or alteration in cellular migration.

**Figure 3:**
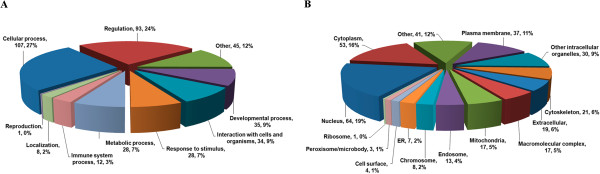
**Pie charts of biological process and cellular component annotation of proteins up-regulated in CHIKF samples.** The biological process **(A)** and cellular component annotation **(B)** of 240 proteins that were up-regulated in CHIKF samples were analyzed by STRAP software.

Many of the 240 proteins identified only in the infected samples are poorly or incompletely characterized (Additional file 4). Categorization of the proteins by biological process using the Software Tool for Rapid Annotation of Proteins (STRAP) bioinformatics suite [30] showed that while the majority of proteins were classed under “regulation” (24%) and “cellular processes” (27%), other proteins mapped to “interaction with cells and organisms” (9%), response to stimulus (7%) and immune system (3%), all of which would be consistent with a response to viral infection (Figure 3 and Additional file 5). Analysis by cellular component showed that the proteins identified mapped to 14 different components, including cytoplasm, nucleus, cytoskeleton, plasma membrane, endosome and peroxisome (Figure 3). A number of proteins link to the processes of cytoskeleton remodeling, cell adhesion, migration, proliferation and vesicle transport, including Plexin-B1, PAK1, Abelson tyrosine-proteinkinase 2, tensin-3, supervillin, WASH complex subunit FAM21A,kinesin-like proteinkIF16B, Dynamin-1 and spartin, suggesting that both cell motility and vesicle trafficking are upregulated in response to infection. Alteration of cytoskeletal proteins of lymphocytes has been reported as a fever associated phenomenon, as has the activation of the ERK1/2 pathway which was also found up regulated in this study [31]. Increases in vesicle trafficking could be associated with increased vesicle formation as a result of increased endocytosis or phagocytosis. Given that CHIKV infection induces apoptosis [16-18] increased phagocytic scavenging of apoptotic bodies is likely to occur.

Surprisingly few proteins mapped to either the apoptosis or autophagy pathways, both of which have been shown to be induced in response to CHIKV infection [16-21]. This result either suggests that these processes are less prominent in actual patient infections that cell culture results would suggest, or that the control, febrile non-CHIKV infection patients have similar processes ongoing and so the analysis does not detect these as differentially regulated.

A total of 34 (14%) of the 240 proteins up-regulated in infected samples were enzymes which mapped to 27 different enzymatic processes. In particular multiple enzymes associated with nucleotide synthesis (purine and pyrimidine metabolism), amino acid synthesis (pyruvate, nitrogen, D-glutamine/glutamate, arginine/proline, alanine/aspartate/glutamate metabolism) and energy production (oxidative phosphorylation, TCA cycle, glycolysis/glucogenesis) were found to be up-regulated. Up-regulation of purine metabolism has recently been reported in Influenza A infection of human airway epithelia cells [32] and remodeling of these and other pathways to facilitate virus production would be consistent with a response to viral infection.

As noted previously, 65 proteins were found only in mild CHIKF cases and a further 46 were found only in severe CHIKF patients. Protein ontology analysis showed remarkably little difference in either the protein biological function or cellular component (Additional file 6: Figure S2). We observed that four calcium regulated genes (KCNMB4, CABIN1, G6B and SPTBN1) were found over-expressed only in mild cases. A further 5 calcium regulated genes (S110A12, CXCR1, ITPR2, PPP3CC and MYLK) were found in both mild and severe CHIKF samples, but that none were found only in severe cases. Calcium (Ca^2+^) is a well known regulator of a number of cellular processes, and while the majority of Ca^2+^ is sequestered, its release can be mediated by inositol phosphates [33] and the receptor for inositol 1,4,5-trisphosphate was found up-regulated in both mild and severe cases, suggesting that calcium mediated signal transduction may play a significant role in CHIKV infection.

Overall however, very little clear differences were seen in the proteins uniquely expressed by either mild or severe cases, given that both the functional and component distribution of the unique proteins were so similar. This could suggest that the course of the disease (mild or severe) is initiated very early in the infection process and that similar but distinct processes are initiated that lead to the distinctive patient disease courses. This is further supported by the much larger number of proteins that are shared between control and mild and control and severe (Figure 2). The 190 proteins that are expressed in both control and mild represent proteins that are down regulated in severe CHIKF, and similarly the 121 proteins shared between control and severe are proteins that are down regulated in mild CHIKF cases. The proteins uniquely up-regulated in mild or severe cases have potential for future development as biomarkers to predict the course of the disease.

Phosphatidylinositol-4,5-bisphosphate 3-kinase, a class 1 phosphatidylinositol 3kinase (PI3K) was found up regulated in mild and severe cases. Phosphatidylinositol-4,5-bisphosphate 3-kinase regulates a number of cellular processes [34] and several other proteins detected as up-regulated also mapped to these pathways, including focal adhesion (Rap guanine nucleotide exchange factor (GEF) 1, myosin light chainkinase, PAK1; p21 protein (Cdc42/Rac)-activatedkinase 1 and MAPK10; mitogen-activated proteinkinase 10, B-raf, talin), T cell receptor signaling (PAK1, PPP3CC and Bcl10), insulin signaling pathway (RAPGEF1, PCK1, MAPK10 and PYGL, B-Raf) and osteoclast differentiation (MCF4, PPP3CC and MAPK10). Other pathways with two other proteins up-regulated include ErbB signaling, HIF signaling, PI3K-AKT signaling, regulation of actin cytoskeleton, Toll like receptor signaling, Natural killer cell mediated cytotoxicity, B cell receptor signaling and chemokine signaling. All of these pathways are consistent with a response to viral infection.

**Figure 4:**
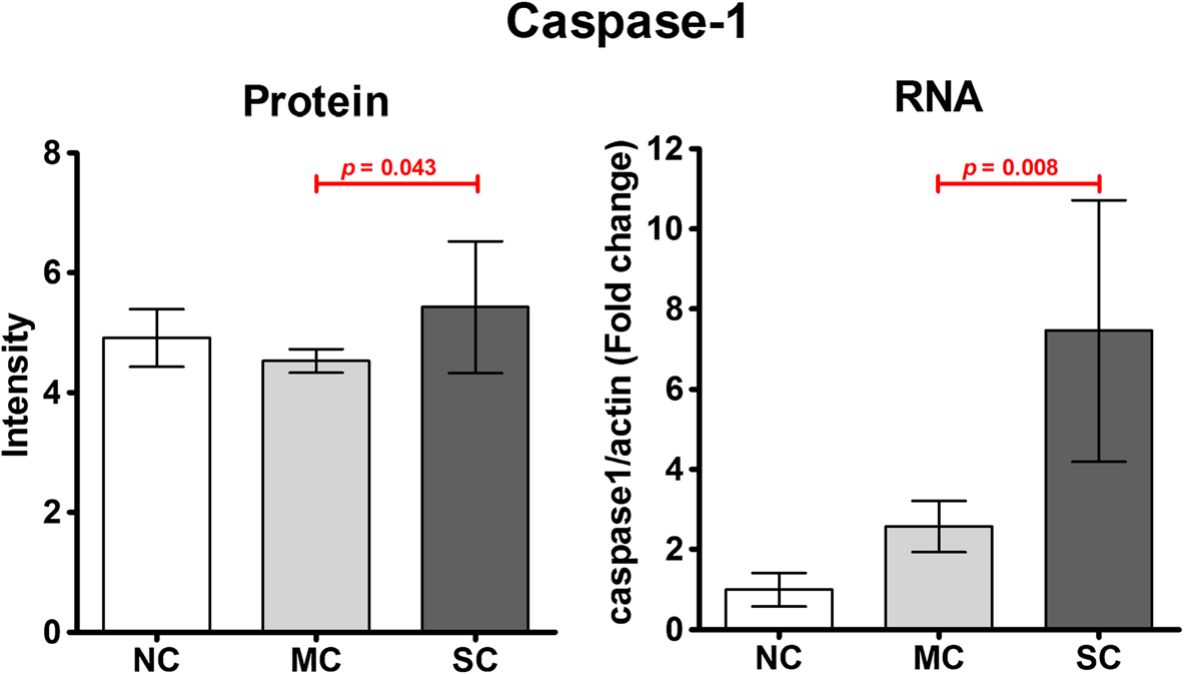
**Quantitation of caspase 1 expression.** The relative expression level of pro-caspase 1 protein was determined from peptide intensity data (after normalization) in the gel slice centered on 45 kDa [as shown in Additional file 1: Figure S1], while expression levels of caspase 1 mRNA were determined by real time RT-PCR.

Toll-like receptor (TLR) 4 was found to be up-regulated in some CHIKF samples. TLR4 is a cell surface expressed [35] pattern recognition molecule that is expressed by monocytes [36]. While TLR4 activation has been primarily characterized in response to LPS, it has also been associated with viral infection [37,38]. TLR4 activation results in the NF-κB mediated up-regulation of pro-IL-1β and pro-IL-18 which are subsequently processed by the inflammasome to the mature inflammatory cytokines IL-Iβ and IL-18 by caspase 1 [39]. There are several different inflammasomes and TLR4 activation leads to the activation of the Nlrp3 inflammasome [39], and NLRP3 was observed to be up-regulated in mild CHIKV cases. A peptide corresponding to caspase 1 was detected in all samples, in different molecular weight gel slices. Analysis of this peptide intensity in the gel slices corresponding to the full length procaspase 1 showed statistically significant differences between the intensity of the peptide between mild CHIKF and severe CHIKF (Figure 4). Using independent samples (2 non-CHIKF, 3 mild CHIKF and 2 severe CHIKF) the expression of the caspase 1 message was quantitated by real time RT-PCR. Results (Figure 4) showed a significant difference in the expression of caspase 1 mRNA between mild and severe CHIKF, which support activation of the inflammasome in response to CHIKV infection.

Evidence is contradictory regarding the levels of IL-Iβ in CHIKV patients. While in an earlier study reported only low levels of IL-Iβ in both mild and severe cases of CHIKF [27], others have reported significantly increased levels of IL-Iβ in severe cases of CHIKF [40], which would be consistent with the results of this study.

## Conclusions

As this study was undertaken on limited clinical material (white blood cells from acute fever patients), we were not able to confirm the results seen here in western analysis. The small sample size (n = 5 for each condition) may additionally result in overestimation of the significance of the results [41]. Trying to re-capitulate the system using infection of purified white blood cells from healthy controls may offer some insights, but it would also tend to exclude proteins whose regulation depend upon complex factors encountered only *in vivo* as previously noted by others [12]. Despite this significant limitation, the results reported here support the direct infection of at least a sub-set of white blood cells, and suggest that further investigation of inflammasome activation and the presence of IL-1β and IL-18 in CHIKF patients is warranted.

## Competing interests

The authors declare that they have no competing interests.

## Authors’ contributions

NW, SU and DRS conceived and designed the study, WP, TT, SU and MT collected and classified samples, NW, AP, SK and SR were responsible for all proteomic work, SK was responsible for real time PCR analysis, NW and DT undertook bioinformatics analysis. NW and DRS wrote the manuscript and all authors contributed to and approved the final version of the manuscript.

## Supplementary Material

Additional file 1: Figure S1 SDS-PAGE of WBC samples. A total of 20 μg of total protein of each of 5 samples of groups non CHIKF (NC), mild CHIKF (MC) and severe CHIKF (SC) were separated by 12.5% SDS-PAGE. Gels were stained with colloidal coomassie blue **(A, B)** and each lane was cut into 13 groups according to size of separated proteins **(C, D)**. Each slice of gel was cut into 1 mm^3^ and these gel plugs were subjected to tryptic digestion. Click here for file

Additional file 2 Full list of structural and non-structural CHIKV peptides detected in CHIKF patients and control (non-CHIKF) patients. Click here for file

Additional file 3 Full list of proteins down regulated in mild and severe CHIKF samples. Click here for file

Additional file 4 Full list of proteins up-regulated in mild and severe CHIKF samples. Click here for file

Additional file 5 Full list of proteins up-regulated in CHIKF patients identified by STRAP analysis as being involved with “Interaction with cells and organisms” (tab 1), “Response to stimulus” (tab 2) and “Immune system” (tab 3). Click here for file

Additional file 6: Figure S2 Pie charts of biological process and cellular component annotation of proteins up-regulated in only mild or severe CHIKF samples. A total of 65 or 46 proteins were up-regulated only in mild or severe CHIKF samples, respectively. The STRAP software was used to annotate these proteins into the biological process **(A, C)** and cellular component **(B, D)**. Click here for file
