# Supplementary figures and images for: Comprehensive proteomic analysis of white blood cells from chikungunya fever patients of different severities
Source: J Transl Med. 2014 Apr 11;12:96. doi: 10.1186/1479-5876-12-96 (PMC4022080; doi:10.1186/1479-5876-12-96)

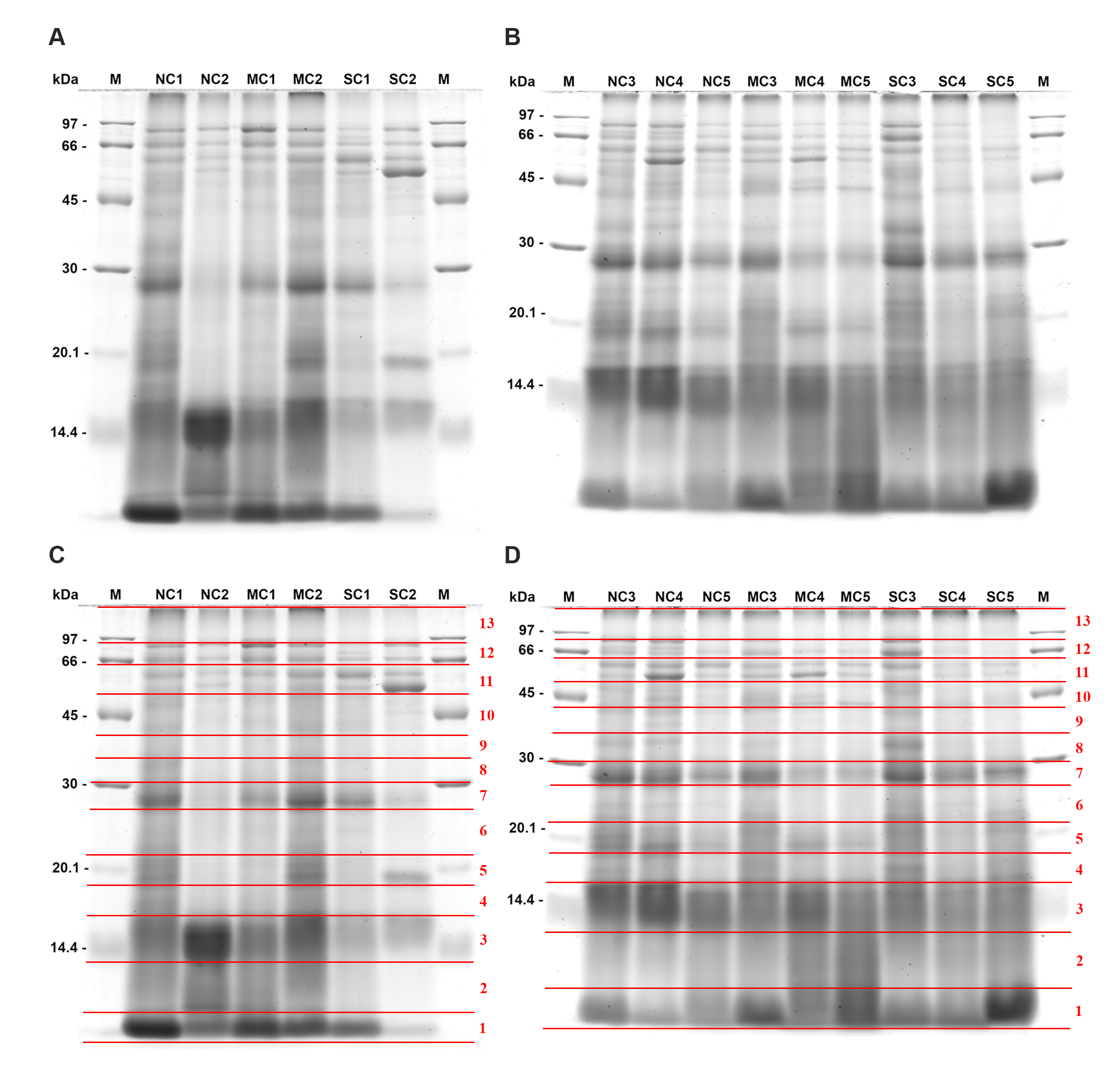

Supplement: Additional file 1: Figure S1 — SDS-PAGE of WBC samples. A total of 20 μg of total protein of each of 5 samples of groups non CHIKF (NC), mild CHIKF (MC) and severe CHIKF (SC) were separated by 12.5% SDS-PAGE. Gels were stained with colloidal coomassie blue (A, B) and each lane was cut into 13 groups according to size of separated proteins (C, D). Each slice of gel was cut into 1 mm3 and these gel plugs were subjected to tryptic digestion. [file 1479-5876-12-96-S1.tiff]

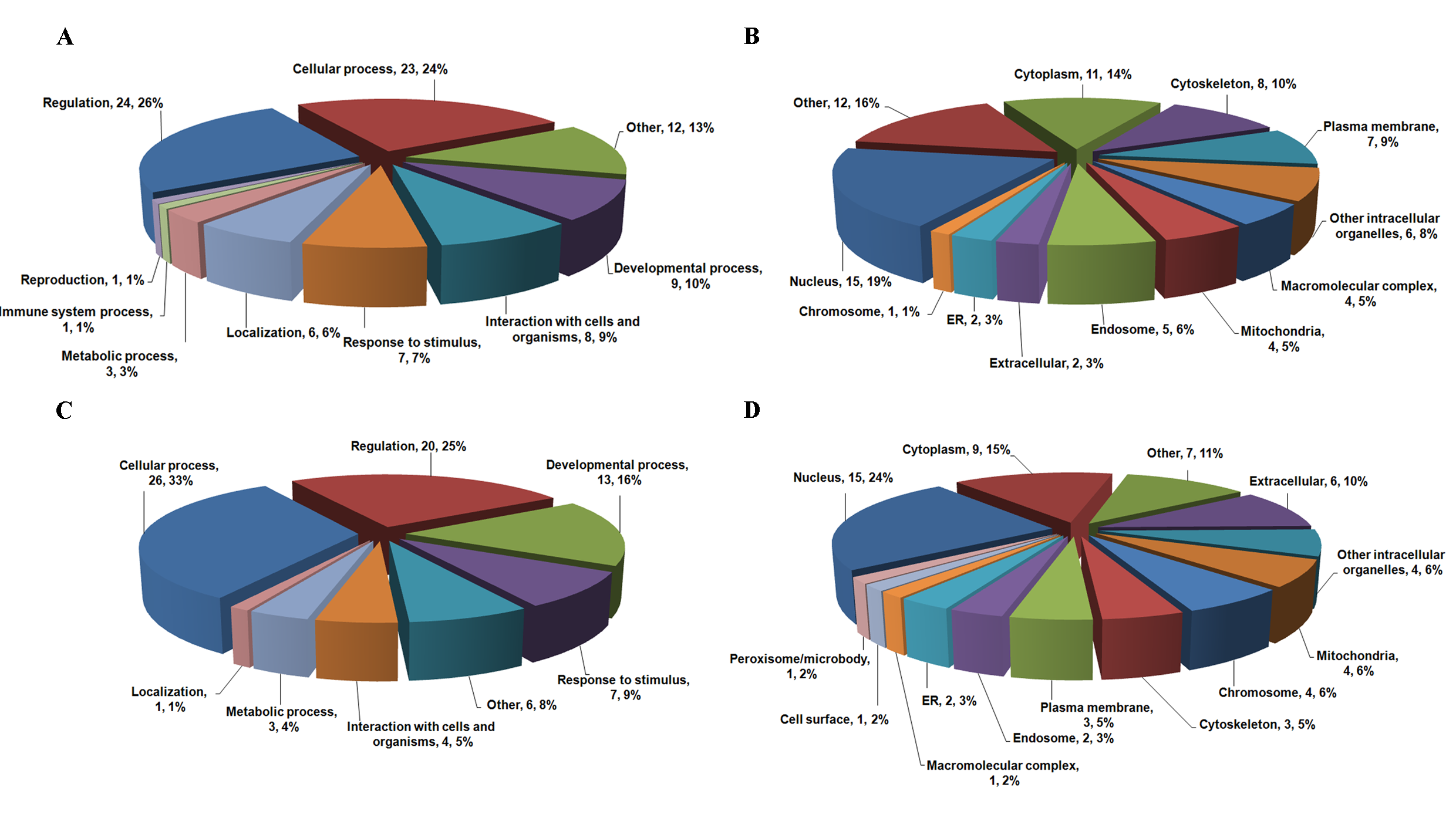

Supplement: Additional file 6: Figure S2 — Pie charts of biological process and cellular component annotation of proteins up-regulated in only mild or severe CHIKF samples. A total of 65 or 46 proteins were up-regulated only in mild or severe CHIKF samples, respectively. The STRAP software was used to annotate these proteins into the biological process (A, C) and cellular component (B, D). [file 1479-5876-12-96-S6.tiff]
